# Multi-Objective Process Optimization of Micro-Milling Titanium Alloy Ti6Al4V for Microgrooves

**DOI:** 10.3390/ma19102142

**Published:** 2026-05-20

**Authors:** Yabo Zhang, Chenyang Wang, Qingshun Bai, Qiqin Zhang, Xin He

**Affiliations:** 1 China Airborne Missile Academy, Luoyang 471009, China; 2 School of Mechanical and Electrical Engineering, Harbin Institute of Technology, Harbin 150001, China; qshbai@hit.edu.cn; 3 School of Mechanical Engineering, Southwest Jiaotong University, Chengdu 610031, Chinahexin@swjtu.edu.cn (X.H.)

**Keywords:** micro-milling, single-factor experiments, orthogonal experiments, multi-objective process optimization, titanium alloy Ti6Al4V microgrooves

## Abstract

High-quality microgrooves obtained in micro-milling titanium alloy Ti6Al4V are still challenging work due to the dependence of burr formation and surface roughness on cutting parameters. In this paper, the systematic analysis of the micro-milling process was conducted to obtain high-quality titanium alloy Ti6Al4V microgrooves, which is based on single-factor experiments, orthogonal experiments, intuitive analysis, range analysis, regression analysis, and multi-objective optimization. The range of factors and factors of orthogonal experiments were determined by single-factor experiments. Orthogonal experiments were conducted with a three-factor three-level design, which regards the total top-burr width and the bottom surface roughness of microgrooves as the response variables, and factors are spindle speed, feed per tooth, and the axial depth of cut. The optimal cutting parameters, which minimize the surface roughness and burr formation, and the main influence factor were determined by intuitive analysis, range analysis, regression analysis, and NSGA-II multi-objective optimization. Simultaneously, high-quality complex microgrooves were achieved with the optimal cutting parameters. The method of systematic experimental design and data analysis in this paper can provide the theoretical guideline and technical support for the processing development of complex parts.

## 1. Introduction

Surface microstructures of components have been widely used in various fields, such as biomedical [1,2] and microfluidic [3,4]. As a type of mechanical micro-cutting method, micro-milling exhibits significant advantages in machining components with complex 3D microstructures [5,6]. It features high-machined surface integrity and dimensional/geometrical accuracy, as well as wide material compatibility and excellent structural compatibility, making it an efficient and high-quality cutting method for surface microstructure [7,8,9]. Many researchers have conducted extensive studies to achieve high-quality microstructure surfaces with diverse features. Zheng et al. [10] conducted vibration-assisted micro-milling experiments on PMMA to investigate the influence of surface texture on microfluidic flow. Wang et al. [11] performed micro-milling of copper layers for microstrip antenna fabrication and explored the effects of cutting parameters. They obtained the high-quality micro-strip antenna with the structure of Archimedean spiral by using the optimal machining parameters. Knap et al. [12] created the microstructure on the aluminum alloy surface by micro-milling and obtained high-quality hydrophobic surfaces on silicone impression material by the replication process of the microstructure on the aluminum alloy surface. Gupta et al. [13] machined different microchannel patterns on stainless steel SS316L using micro-milling and achieved high-quality microchannels by removing burrs through electro polishing processes. Guckenberger et al. [14] highlighted that micro-milling is a highly efficient method for ultra-rapid prototyping of plastic microfluidic devices, which can be further utilized for cell culture applications. Geng et al. [15] conducted micro-milling experiments on poly(methyl methacrylate) (PMMA) surfaces and investigated the influence of tool inclination on surface roughness. Wang et al. [16] constructed a hierarchical micro/nano-topography on the titanium alloy surface through micro-milling followed by an alkali-hydrothermal treatment, and investigated the effect of surface modification on the biocompatibility of implants. The results indicated that the micro/nano-textured surface significantly promotes the adhesion and differentiation of MC3T3 osteoblasts. For surface microstructures, the complex surface microstructures depend on the single microgrooves. Therefore, a comprehensive investigation of the cutting process for the single microgroove is essential to obtain high-quality complex surface microstructures by micro-milling.

Titanium alloy Ti6Al4V has found widespread applications in the biomedical and aerospace industries due to its exceptional properties, including low density, high-temperature stability, excellent corrosion resistance, and superior biocompatibility [17,18,19,20]. To obtain the high-quality titanium alloy Ti6Al4V microstructure, many researchers have conducted extensive studies on the related cutting process. Pratap et al. [21] investigated the influence of surface micro-texture configurations—specifically micro-dimples and micro-grid patterns—on the tribological performance of titanium alloy Ti6Al4V fabricated via ball-end micro-milling. It was found that the micro-grid surface exhibited a lower coefficient of friction. Du et al. [22] conducted investigations of micro-milling micro-texture array on additively manufactured NiTi alloy surfaces. Their results confirmed that micro-milling is an effective method to produce high-quality microgrooves and micro-pillar arrays on such surfaces. Zou et al. [23] employed both a single micro-milling process and a hybrid approach combining picosecond laser ablation and micro-milling to fabricate U-shaped grooves. Their results demonstrated that pre-ablation of the titanium alloy Ti6Al4V, followed by micro-milling, significantly enhances the surface quality of U-shaped grooves. Xia et al. [24] obtained high-aspect-ratio microgrooves on titanium alloy Ti6Al4V surfaces by laser-induced oxidation-assisted micro-milling (LOMM). Compared with conventional micro-milling, LOMM exhibits lower cutting forces, reduced tool wear, and improved surface quality. However, micro-milling is not merely a simple scale-down of macro-milling. Several factors—such as machine dynamics, tool material, cutting environment, and the workpiece’s microstructure and mechanical properties—exert a more pronounced influence on surface quality at the microscale [25,26,27]. Therefore, it is essential to systematically investigate the micro-milling process of titanium alloy Ti6Al4V microgrooves and identify optimal cutting parameters for achieving the high surface quality.

The primary surface quality characteristics in micro-milling microgrooves are micro-burrs and surface roughness. The formation of micro-burrs can damage the functionally related performance and deteriorate the surface quality in micro-milling microgrooves. At the same time, small burr size and surface roughness value mean a high surface quality of the microgrooves. However, surface roughness and burr size are strongly dependent on cutting parameters. When the surface roughness value is minimized under specific cutting conditions, burr size may increase significantly, potentially compromising surface quality. Therefore, achieving high-quality microgrooves in micro-milling titanium alloy Ti6Al4V remains a significant challenge. In the present study, systematic micro-milling experiments and data analysis methods, such as single-factor experiments, orthogonal experiments, intuitive analysis, range analysis, regression analysis, and NSGA-II multi-objective optimization, were conducted to explore the optimal cutting parameters for obtaining high-quality titanium alloy Ti6Al4V microgrooves by micro-milling. In the micro-milling experiments, the bottom surface roughness and the total top-burr width of the microgrooves were selected as response variables, while spindle speed, feed per tooth, and axial depth of cut were considered as variable factors. The systematic experimental design and data analysis method proposed in this paper can provide theoretical guidance and technical support for the exploration of new materials manufacturing processes.

## 2. Methodology

### 2.1. Experimental Procedure

The objective of the experimental design is to minimize the number of tests while achieving improved results. In general, experimental investigation of metal cutting focuses on four key aspects: (1) identification of the critical factors influencing the response variables; (2) analysis of the influence laws of these factors on the response variables; (3) determination of optimal processing parameters; and (4) verification of the optimal parameter sets. To identify the key factors influencing surface quality and optimize cutting parameters in micro-milling titanium alloy Ti6Al4V microgrooves, a hybrid experimental approach combining single-factor and orthogonal designs was implemented. Multiple analytical techniques—namely intuitive analysis, range analysis, regression analysis, and NSGA-II multi-objective optimization—were applied to derive multi-perspective insights from the experimental data.

**Figure 1:**
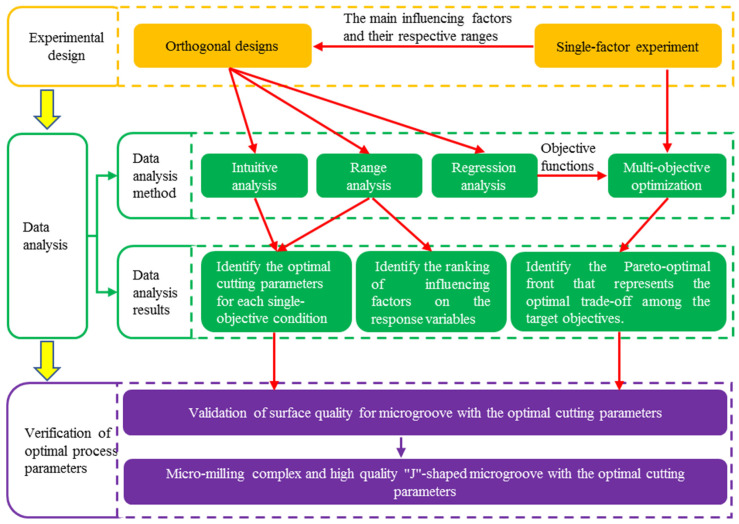
Flowchart of the experimental procedure.

Figure 1 presents the flowchart of the experimental procedure for this paper. This experimental procedure includes three parts: experimental design, data analysis and verification of optimal process parameters. Single-factor and orthogonal designs were adopted in the part of the experimental design. The main influencing factors and their respective ranges were identified through single-factor experiments. Meanwhile, it was used as input for the orthogonal experiments. Based on this, the factors and their upper and lower limits of the orthogonal experiment were determined. Specifically, cutting parameters were selected as the experimental factors, and their feasible ranges were identified by the results of the single-factor experiments. Orthogonal experiments were conducted to obtain experimental data, which includes surface roughness and top-burr width under varying cutting parameters. The data analysis section includes data analysis methods and data analysis results. The data analysis methods of orthogonal experiments were intuitive analysis, range analysis and regression analysis. Based on intuitive analysis and range analysis, the optimal cutting parameters for each single-objective condition were identified. In addition, range analysis was used to identify the ranking of influencing factors on the response variables. Based on regression analysis, nonlinear regression models for surface roughness and burr size were developed. Based on the nonlinear regression model established through regression analysis and the upper and lower bounds of cutting parameters identified from single-factor experiments, the multi-objective optimization of cutting parameters was conducted using the NSGA-II algorithm, yielding a Pareto-optimal front that represents the optimal trade-off among the target objectives. In the part of verification of optimal processing parameters, based on the multi-objective optimization results, a set of promising cutting parameters was selected and validated through single microgroove machining experiments. The resulting data were compared with the orthogonal experimental results to determine the final optimal cutting parameters. The high-quality complex “Ji”-shaped microgrooves were fabricated with the optimized cutting parameters.

### 2.2. Experimental Setups

**Figure 2:**
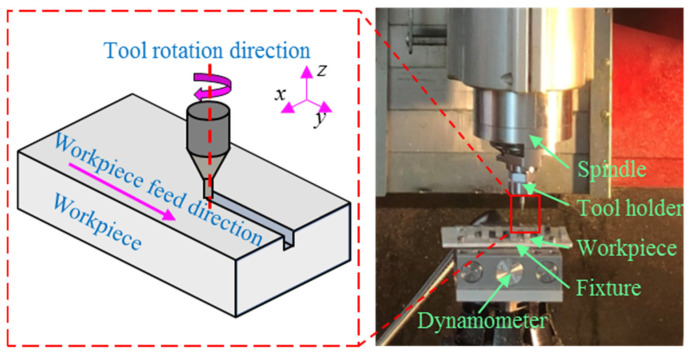
Setup of micro-milling experiments and schematic diagram of the slot micro-milling [28].

**Figure 3:**
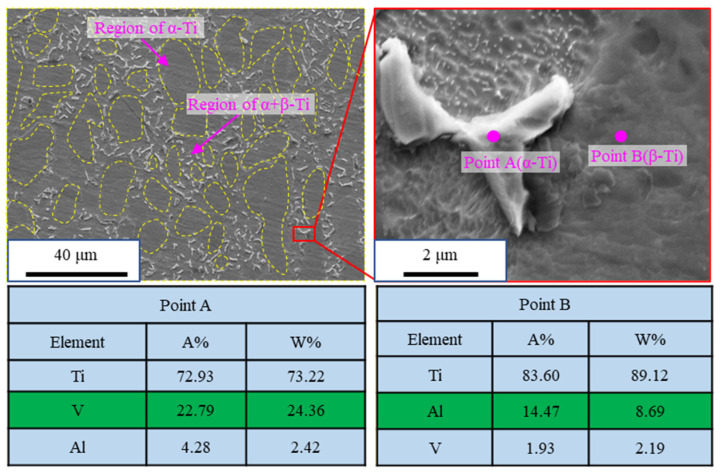
Metallographic micrographs and EDS analysis of the as-received Ti-6Al-4V alloy [28].

**Figure 4:**
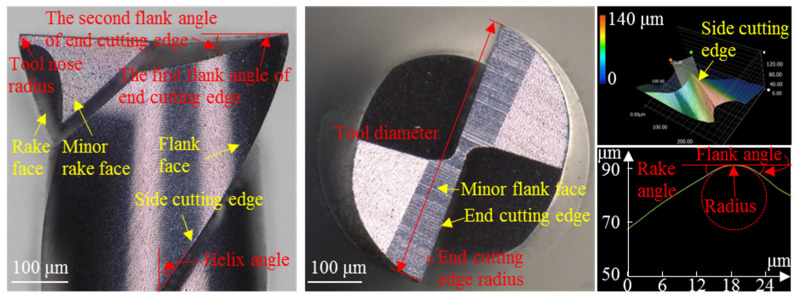
Optical images of the micro-milling tool.

Micro-milling experiments were conducted on the Kern Evo five-axis ultra-precision micro-milling machine (Kern Microtechnik GmbH, Eschenlohe/Murnau, Germany). Figure 2 shows the machine setup and the schematic illustration of the micro-milling process. The tool was mounted in the spindle using the tool holder. The Kern Evo five-axis ultra-precision micro-milling machine features a tool magazine, automatic tool changer, and laser tool alignment device, enabling automatic tool change, alignment, and runout measurement. Runout values measured by the laser device were fed into the machine program to calculate tool radius compensation for high-precision machining. The workpiece material was titanium alloy Ti6Al4V, supplied by Baoti Group (BAOTI Group Co., Ltd., Baoji City, China). Rectangular blocks of titanium alloy Ti6Al4V were used as specimens with the dimensions of 20 mm × 10 mm × 5 mm^3^, and the top surface served as the machining area. The specimens were bonded to the fixture using KH502 quick-drying adhesive. Figure 3 presents the microstructure and energy dispersive X-ray spectroscopy (EDS) analysis of titanium alloy Ti6Al4V specimens. It can be observed that titanium alloy Ti6Al4V is a kind of multiphase alloy, consisting of the α-Ti matrix phase and the β-Ti phase. Table 1 presents the mechanical properties of titanium alloy Ti6Al4V. In the experiments, commercial TiAlN-coated micro-milling tools (NS MX235, NS TOOL CO., LTD, Tokyo, Japan) were employed with a diameter of 500 μm. Figure 4 shows the optical images of the micro-milling tool, captured using the VHX-1000 extended depth of field (EDF) microscope (Keyence corporation, Osaka, Japan). The corresponding tool parameters are summarized in Table 2.

Material removal mechanism in micro-milling titanium alloy Ti6Al4V microgrooves is discussed in detail in Refs. [28,30], especially in burr formation and surface roughness. The single-factor experiment was conducted to explore the effect of cutting parameters on the surface morphology and burr formation in Refs. [28,30]. It can be seen that cutting parameters significantly affect surface roughness and burr size in micro-milling titanium alloy Ti6Al4V. To systematically investigate the effects of these parameters, the three-factor, three-level orthogonal experimental design was employed in this study. The spindle speed, feed per tooth, and axial depth of cut were selected as the input factors, while the top burr width and the bottom surface roughness value of the microgroove were taken as the response variables. The factor levels and experimental settings are detailed in Table 3. According to the single-factor experimental results in Refs. [28,30], surface roughness and burr height effectively reflect the variation characteristics of micro-milling across the following cutting parameter ranges: spindle speed 22,000–34,000 r/min, feed per tooth 0.3–1.1 μm/t, and axial depth of cut 20–40 μm. Therefore, the experimental levels for spindle speed, feed per tooth, and axial depth of cut were set within the ranges of 20,000–34,000 r/min, 0.1–1.0 μm/t, and 10–40 μm, respectively. In micro-milling, the cutting process can be divided into two stages. In the first stage, the workpiece surface was smoothed to remove the surface oxide layer by a titanium alloy-specific cutting tool with a diameter of 3 mm. The cutting parameters were set at spindle speed 10,000 r/min, feed per tooth 0.5 μm/t, and axial depth of cut 50 μm. In the second stage, micro-milling was performed on the as-machined surface by a micro-milling tool with a diameter of 500 μm. The micro-milling length of the microgroove is 5 mm with various levels of cutting parameters. All microgrooves were machined without any coolant or lubricant. Each level of cutting parameters was repeated twice to minimize the influence of random errors. Moreover, the tool wear has a significant influence on surface formation, especially in burr size and surface roughness. The effect of tool wear on surface formation and cutting force is discussed in [25,31,32,33,34]. To eliminate the effect of tool wear on the results, the new micro-milling tool was used in each repetition.

**Figure 5:**
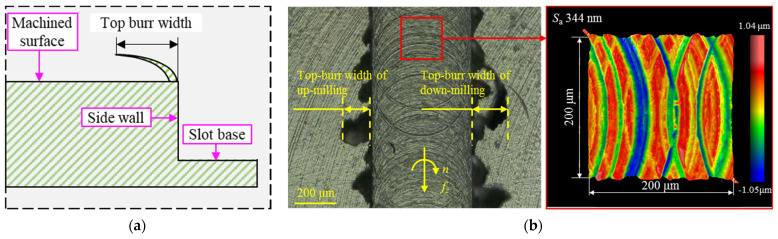
Measurement illustration of the top burrs and surface roughness of the microgroove. (**a**) Schematic diagram of top burr width (**b**) measurement location for top burrs and surface roughness of the microgroove.

The top burr size was measured with the VHX-1000 EDF microscope (KEYENCE International, Mechelen, Belgium). The top-burr width of the microgroove is defined as a horizontal length of burr from the microgroove sidewall, as shown in Figure 5a. To minimize measurement errors and ensure data reliability, the top burr width was measured at three different positions along the microgroove where the burr height was uniform, and the average value was taken as the mean top-burr width. Meanwhile, the top burr width varies significantly between up-milling and down-milling due to the different burr formation mechanism according to the reference [30]. In this study, the mean total top-burr width *b*_w_, defined as the sum of the mean top-burr widths from up-milling and down-milling, is adopted as the response variable of the orthogonal experiment. The bottom surface roughness of the microgroove was measured with the white-light interferometer (Zygo NewView 8300, Zygo, Middlefield, CT, USA). As reported in reference [28], the bottom surface roughness is non-uniform along the width of the microgroove. However, the surface roughness in the central region of the microgroove is relatively uniform, making it suitable for quantitative analysis of surface roughness. Therefore, the bottom surface roughness value *S*_a_ was measured within a 200 × 200 μm^2^ region at the center of the microgroove and used as the response variable in this study, as shown in Figure 5b. To minimize measurement errors, the bottom surface roughness *S*_a_ was measured at three different regions along each microgroove, and the average value was taken as the mean bottom surface roughness of the microgroove. This mean value was adopted as the response variable in the orthogonal experiment. The results of the orthogonal experiments are summarized in Table 4.

## 3. Results and Discussion

### 3.1. Intuitive Analysis and Range Analysis

The range analysis results for the mean total top-burr width *b*_w_ and the mean bottom surface roughness *S*_a_ in micro-milling titanium alloy Ti6Al4V are summarized in Table 5 and Table 6. It can be seen from Table 5 that the axial depth of cut *a_p_* has the most significant effect on the mean total top-burr width *b*_w_, followed by spindle speed *n* and feed per tooth *f*_t_, with the range values of 78.83, 57.22, and 41.27, respectively. This indicates that controlling axial depth of cut is critical for minimizing burr formation in micro-milling titanium alloy Ti6Al4V. Meanwhile, Table 6 reveals that feed per tooth *f*_t_ has the most significant effect on the mean bottom surface roughness *S*_a_, followed by spindle speed *n* and axial depth of cut *a_p_*, with the range values of 160.55, 70.89, and 43.78, respectively. This indicates that feed per tooth is the dominant factor influencing surface roughness in micro-milling titanium alloy Ti6Al4V, highlighting the importance of precise control over this parameter.

**Figure 6:**
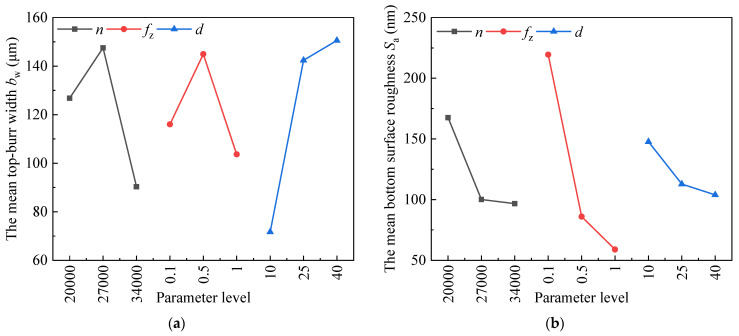
Main effects plot of the response variables: (**a**) the mean top-burr width *b*_w_, (**b**) the mean bottom surface roughness *S*_a_.

Figure 6 presents the main effect plots of the mean total top-burr width *b*_w_ and the mean bottom surface roughness *S*_a_, revealing the influence of cutting parameters. When the response variable is the mean total top-burr width *b*_w_, a lower value is desirable. The optimal cutting parameters derived from the intuitive analysis indicate that Number 1 yields the minimum mean total top-burr width *b*_w_ with the spindle speed 20,000 r/min, feed per tooth 0.1 μm/t, and axial depth of cut 10 μm, as shown in Table 4. In contrast, Figure 6a indicates that the best combination for minimum mean total top-burr width *b*_w_ is different with spindle speed 20,000 r/min, feed per tooth 1 μm/t, and axial depth of cut 10 μm, in terms of the optimal cutting parameters determined by the range analysis. Meanwhile, when the response variable is the mean bottom surface roughness *S*_a_, a lower value is preferred. The optimal cutting parameters derived from the intuitive analysis show that Number 6 yields the minimum mean bottom surface roughness *S*_a_ with the spindle speed 27,000 r/min, feed per tooth 1 μm/t, and axial depth of cut 10 μm, as shown in Table 4. However, range analysis identifies a different optimal combination for the minimum mean bottom surface roughness *S*_a_ with spindle speed 34,000 r/min, feed per tooth 1 μm/t, and axial depth of cut 40 μm as shown in Figure 6b. In summary, the intuitive analysis and range analysis yield different optimal cutting parameter combinations with the same response variable. At the same time, the mean total top-burr width *b*_w_ and the mean bottom surface roughness *S*_a_ are cutting parameter dependent. When the response variables are the mean total top-burr width *b*_w_ and the mean bottom surface roughness *S*_a_, respectively, the optimal cutting parameter sets are different. Therefore, it is unrealistic to simultaneously achieve the minimum mean total top-burr width *b*_w_ and the minimum mean bottom surface roughness *S*_a_ with the same cutting parameters. In order to identify the more favorable cutting parameter combinations, regression analysis and multi-objective optimization are performed on the orthogonal experimental data, providing significant guidance for determining the optimal machining process.

### 3.2. Regression Analysis and Multi-Objective Process Optimization

To further clarify the relationships between the response variable —the mean total top-burr width *b*_w_, the mean bottom surface roughness *S*_a_ and cutting parameters—spindle speed *n*, feed per tooth *f*_t_ and axial depth of cut *a*_p_, the multivariate nonlinear regression analysis was performed on the orthogonal experimental data. The obtained nonlinear regression model equations are shown in Equations (1) and (2), respectively.

(1) $$\begin{array}{c} b_{w}=-643.8+45.72n+159.3f_{z}+14.76d-2.145n\cdot f_{z}-0.1692n\cdot d\\ -0.7954n^{2}-122.7{f_{z}}^{2}-0.1556d^{2} \end{array}$$

(2) $$\begin{array}{c} S_{a}=937.4-38.63n-1085f_{z}+1.726d+14.73n\cdot f_{z}-0.3819n\cdot d\\ \;+0.653n^{2}+421.8{f_{z}}^{2}+0.1723d^{2} \end{array}$$

**Figure 7:**
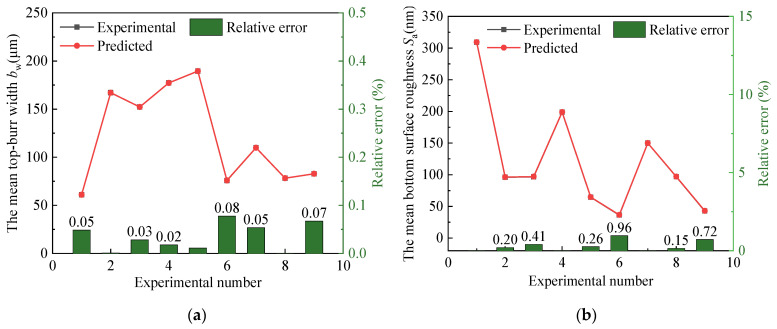
Experimental values, predicted values and the relative error: (**a**) the mean top-burr width *b*_w_ of the microgroove, (**b**) the mean bottom surface roughness *S*_a_ of the microgroove.

To verify the adequacy of the nonlinear regression model equations, the coefficient of determination (*R*^2^) was calculated. The nonlinear regression models for both the mean total top-burr width *b*_w_ value exhibit a high *R*^2^ value of 0.99, indicating outstanding predictive accuracy and strong consistency between predicted and experimental results. Meanwhile, Figure 7 presents the experimental values and predicted values of the mean total top-burr width *b*_w_ and the mean bottom surface roughness *S*_a_, and the absolute values of relative error. It can be seen that the fitting values show excellent agreement with the experimental measurements, with maximum prediction errors of only 0.04% for *b*_w_ and 0.96% for *S*_a_. These low errors confirm the high accuracy and reliability of the developed nonlinear regression models. Therefore, the nonlinear regression model developed in this study can be well fitted to the real data of the mean total top-burr width *b*_w_ and the mean bottom surface roughness *S*_a_ with respect to cutting parameters.

It can be seen from intuitive analysis and range analysis that it is impossible to simultaneously minimize both the mean total top-burr width *b*_w_ and the mean bottom surface roughness *S*_a_ with the same cutting parameters. Therefore, no single optimal solution exists, and there is a trade-off between minimizing the mean total top-burr width *b*_w_ and mean bottom surface roughness *S*_a_. Meanwhile, multi-objective optimization generates a Pareto-optimal solution set, offering a range of trade-off options for balancing competing performance criteria, and is well-suited for determining optimal cutting parameter combinations. In this study, the Non-dominated Sorting Genetic Algorithm II (NSGA-II), a multi-objective optimization algorithm, was employed to determine the optimal cutting parameter combinations that minimize both top-burr width and surface roughness. The NSGA-II is an advanced multi-objective optimization algorithm that integrates the evolutionary mechanism of the traditional genetic algorithm with the Pareto optimality concept, enabling efficient identification of non-dominated solutions. The algorithm follows a process similar to traditional intelligent optimization: initializing a population, evaluating response values, comparing individuals to identify the best solution, updating positions iteratively, and repeating until convergence or maximum iterations. The fundamental differences among various intelligent optimization algorithms lie in their individual position update rules and the criteria used to identify the global optimum. The performance of intelligent optimization algorithms is significantly influenced by the population size and number of iterations. In general, increasing the population size and the number of iterations enhances solution accuracy at the expense of longer computational time. Therefore, an appropriate combination of population size and number of iterations must be carefully selected to balance the solution accuracy and efficiency.

To implement multi-objective optimization with intelligent algorithms, the objective functions, optimization objective, optimizing variables, constraints, and the upper and lower bounds of the optimizing variable must be clearly defined, forming the foundation of the optimization model. In this study, orthogonal experiments provide the original data as shown in Table 4. Equations (1) and (2) are regarded as the objective functions, and the optimization objectives are the minimum mean total top-burr width *b*_w_ and the minimum mean bottom surface roughness *S*_a_. The optimizing variables are spindle speed, feed per tooth and axial depth of cut. The constraints are that *b*_w_ > 0 and *S*_a_ > 0. The single-factor experimental results in Refs. [28,30] revealed that both the top-burr width and bottom surface roughness exhibit inflection points with respect to micro-milling parameters. This suggests that the parameter ranges—22,000–34,000 r/min for spindle speed, 0.1–1.1 μm/t for feed per tooth, and 20–40 μm for axial depth of cut—are well-suited as the initial bounds of the multi-objective optimization, ensuring the search space covers the region of optimal performance. Finally, the upper and lower bounds for the three optimization variables were determined with consideration of both machine tool capabilities and the results from the single-factor micro-milling experiments, as summarized in Table 7.

**Figure 8:**
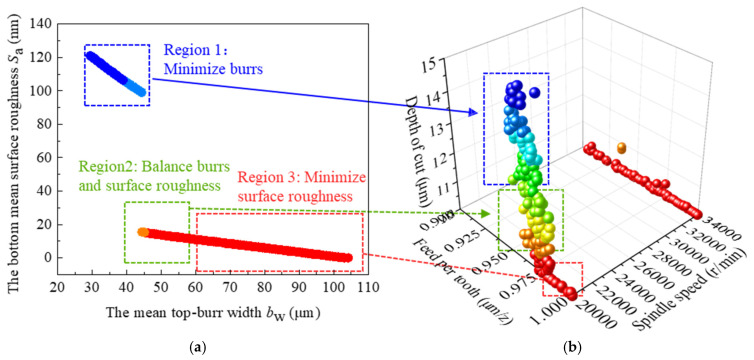
Optimal results of NSGA-II (**a**) Pareto front of optimal response variable (**b**) optimal solution in variable domain.

To balance computational accuracy and efficiency, a population size of 250 and a maximum of 500 generations was adopted for multi-objective optimization in this study. In contrast to single-objective optimization, which seeks a single optimal solution, multi-objective optimization generates a Pareto-optimal solution set, offering a range of trade-off options for balancing competing performance criteria in practical applications. The Pareto frontier of the non-dominated solutions for two objective functions—minimizing mean total top-burr width *b*_w_, and minimizing mean bottom surface roughness *S*_a_—is presented in Figure 8, along with a three-dimensional visualization of the variable space corresponding to these solutions. As shown in Figure 8a, the Pareto frontier clearly demonstrates a trade-off between minimizing *b*_w_ and *S*_a_, and no single set of cutting parameters can simultaneously minimize both responses. Meanwhile, any solution of the non-dominated solutions is not strictly superior to the others, and any solution within the non-dominated solutions can be considered acceptable. According to the requirements of the research objectives, the non-dominated solution set in Figure 8a can be divided into three distinct regions. There are marked as “minimize burrs (region 1)”, “balance burrs and surface roughness (region 2)”, and “minimize surface roughness (region 3)”. The final selection of the optimal solution is determined by the specific machining objectives and the relative weights assigned to the competing performance criteria, reflecting the trade-offs inherent in the optimization process. When surface roughness is prioritized over burr size, Region 1 offers the best trade-off for surface quality. Conversely, when burr size is the primary concern, Region 3 provides the most favorable solution. For balanced performance, Region 2 is recommended as it offers a well-balanced compromise between the two objectives.

### 3.3. Process Optimization Validation of Titanium Alloy Ti6Al4V Groove Structures

**Figure 9:**
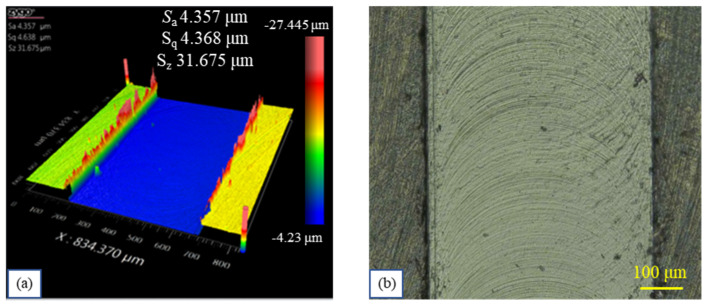
Images of the microgrooves under the optimal parameters (**a**) white-light interferometer image, (**b**) EDF image.

To simultaneously achieve low mean total top-burr width *b*_w_ and the mean bottom surface roughness *S*_a_, the parameter set from Region 2 in Figure 8—comprising the spindle speed 20,000 r/min, the feed per tooth 1 μm/t, and the axial depth of cut 11 μm—is selected as the optimal solution, offering a well-balanced compromise between surface roughness and burr suppression. Micro-milling experiments were conducted under the selected optimal cutting parameters, and Table 8 presents the experimental results of the total top-burr width and bottom surface roughness of the microgroove. The mean total top-burr width *b*_w_ and the mean bottom surface roughness *S*_a_ are 34.46 μm and 25 nm, respectively, which are smaller than the results of the orthogonal experiment, as shown in Table 4. At the same time, Figure 9a,b present the images of the white-light interferometer and EDF for the microgrooves under the selected optimal cutting parameters. It can be seen that both the up-milling and down-milling of the microgroove exhibit a small top-burr size, and no significant defects can be found on the bottom of the microgroove. Therefore, the microgroove exhibits excellent surface quality with the selected optimal cutting parameters.

**Figure 10:**
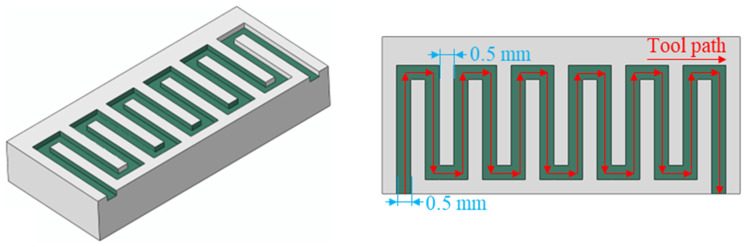
Three-dimensional and two-dimensional schematic diagrams of the “J” shaped groove structure.

**Figure 11:**
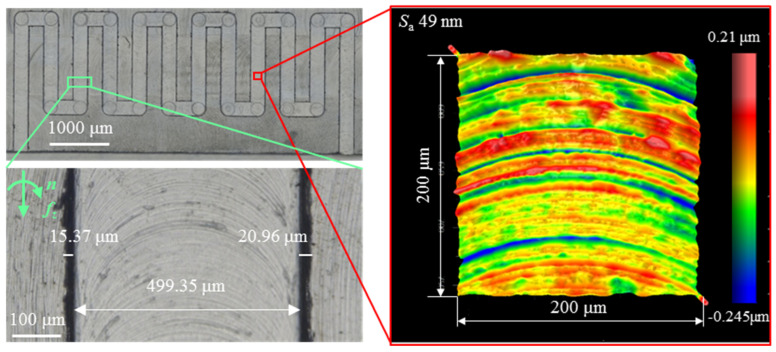
Surface morphologies of “J” shaped groove structure.

In micro-milling, the fabrication of complex microgroove structures is based on the single microgroove, and process exploration of the single microgroove can provide guidance for obtaining high-quality complex microgrooves. In contrast to the micro-milling single microgroove, tool wear has a more serious influence on surface quality for complex microgroove structures due to the longer micro-milling distance than the single microgroove. Based on the above analysis, a “J”-shaped microgroove was obtained on the titanium alloy Ti6Al4V by micro-milling with the selected optimized parameters—spindle speed 20,000 r/min, feed per tooth 1 μm/t, and axial depth of cut 11 μm. Figure 10 presents the three-dimensional and two-dimensional schematic diagrams of the “J” shaped microgroove. The microgroove width is equal to the tool diameter (500 μm), and the horizontal spacing between adjacent microgrooves is designed as 0.5 mm to ensure sufficient material between microgrooves and prevent tool interference. As illustrated in Figure 11, the bottom surface roughness and top-burr width of the “J”-shaped microgroove were measured at a randomly selected location along the central region of the microgroove, with the obtained total top-burr width 36.33 μm, bottom surface roughness 49 nm, and microgroove width 499.35 μm. The top burr width and bottom surface roughness of the “J”-shaped microgroove are slightly higher than those obtained in the single microgroove, which may be attributed to tool wear and machine tool instability—random factors inherent in micro-milling.

## 4. Conclusions

In summary, a systematic process optimization of micro-milling microgrooves for titanium alloy Ti6Al4V was conducted to identify the optimal cutting parameters and the key factors influencing surface quality. This was achieved through a comprehensive experimental design and data analysis method, including single-factor experiments, orthogonal experiments, intuitive analysis, range analysis, regression analysis, and multi-objective optimization. In the experimental design, cutting parameters were regarded as the factors, and the total top-burr width and the bottom surface roughness of microgrooves as the response variables with the optimal objective of minimizing the mean total top-burr width *b*_w_ and the minimum mean bottom surface roughness *S*_a_. The method of systematic experimental design and data analysis for this paper can provide the theoretical guideline and technical support for the processing development of complex parts. The main conclusions are as follows.

1. A comprehensive methodology of exploring the micro-milling process for microgroove was proposed, integrating single-factor experiments, orthogonal experiments, intuitive analysis, range analysis, regression analysis, and NSGA-II multi-objective optimization. Single-factor experiments were conducted to identify the factors and the range of factors. Experimental data were obtained by orthogonal experiments. Range analysis was employed to identify the relative importance of influencing factors. Range analysis revealed the relative importance of each influencing factor. The optimal process parameters were determined through a comprehensive analysis of the orthogonal experimental results, integrating intuitive analysis, regression analysis, and multi-objective optimization.
2. The range analysis of the orthogonal experimental results reveals that the influence order of cutting parameters on the total top-burr width of titanium alloy Ti6Al4V microgrooves is axial depth of cut, spindle speed, and feed per tooth. For the bottom surface roughness of the microgroove, the ranking of influencing factors is feed per tooth, axial depth of cut, and spindle speed.
3. Based on the results of multi-objective process optimization and experimental validation, the optimal cutting parameters for micro-milling titanium alloy Ti6Al4V microgrooves are identified as spindle speed 20,000 r/min, feed per tooth 1 μm/t, and axial depth of cut 11 μm. With these parameters, the “J”-shaped microgroove was successfully fabricated with the bottom surface roughness of 49 nm, total top-burr width 36.33 μm.

## Figures and Tables

**Table 1:** Mechanical properties of titanium alloy Ti6Al4V [29].

Phase Transition Temperature Tβ/°C	Elastic Modulus/GPa	Yield Stress/MPa	Tensile Stress/MPa	Hardness/HV	Bending Rigidity%EI	Fracture Toughness/MPa·mm^1/2^
995	110–140	800–1100	900–1200	300–400	13–16	33–110

**Table 2:** Structure parameters of micro-milling cutters [28].

Structure Parameters	Value	Structure Parameters	Value
Diameter (μm)	~500	Rake angle of side edge (°)	32.69
Helix angle (°)	35	Flank angle of side edge (°)	20.14
Side cutting edge radius (μm)	3.81	Rake angle of end cutting edge (°)	0
End cutting edge radius (μm)	0.77	The first flank angle of the end cutting edge (°)	11.65
Tool nose radius (μm)	0.83	The second flank angle of the end cutting edge (°)	19.8

**Table 3:** Parameters and levels assigned for the experiments.

Input Factors	Level-1 (Low Level)	Level 0	Level 1 (High Level)
Spindle speed *n* (r/min)	20,000	27,000	34,000
Feed per tooth *f*_t_ (μm/t)	0.1	0.5	1
Axial depth of cut *a_p_* (μm)	10	25	40

**Table 4:** Orthogonal experiment design and results with three factors and three levels.

Number	Encoded Value			Actual Value			Response Value	
	A	B	C	*n* (r/min)	*f*_t_ (μm/t)	*a_p_* (μm)	The Mean Total Top-Burr Width *b*_w_ (μm)	The Mean Bottom Surface Roughness *S*_a_ (nm)
1	−1	−1	−1	20,000	0.1	10	61.023	309.33
2	−1	0	0	20,000	0.5	25	167.117	96.33
3	−1	1	1	20,000	1	40	152.263	97.00
4	0	−1	0	27,000	0.1	25	177.213	199.00
5	0	0	1	27,000	0.5	40	189.493	64.67
6	0	1	−1	27,000	1	10	75.893	36.67
7	1	−1	1	34,000	0.1	40	109.877	150.00
8	1	0	−1	34,000	0.5	10	78.220	97.00
9	1	1	0	34,000	1	25	82.853	43.00

**Table 5:** Range analysis table of the mean top-burr width, *b*_w,_ for the microgroove.

Input Factors	Level-1	Level 0	Level 1	Max-Min	Rank
Spindle speed *n* (r/min)	126.8	147.53	90.32	57.22	2
Feed per tooth *f*_t_ (μm/t)	116.04	144.94	103.67	41.27	3
Axial depth of cut *a*_*p*_ (μm)	71.71	142.39	150.54	78.83	1

**Table 6:** Range analysis table of the mean bottom surface roughness *S*_a_ for the microgroove.

Input Factors	Level-1	Level 0	Level 1	Max-Min	Rank
Spindle speed *n* (r/min)	167.55	100.11	96.67	70.89	2
Feed per tooth *f*_t_ (μm/t)	219.44	86	58.89	160.55	1
Axial depth of cut *a_p_* (μm)	147.67	112.78	103.89	43.78	3

**Table 7:** Upper and lower limits of optimization variable values.

Optimization Variable	Spindle Speed *n* (r/min)	Feed per Tooth *f*_t_ (μm/t)	Axial Depth of Cut *a_p_* (μm)
Upper limit	34,000	1	40
Lower limit	20,000	0.1	10

**Table 8:** Experimental results with the optimal parameters.

Response Variables	Measuring Point 1	Measuring Point 2	Measuring Point 3	Mean
Total top-burr width *b*_w_ (μm)	27.92	39.85	35.62	34.46
Surface roughness *S*_a_ (nm)	29	22	24	25

## Data Availability

The original contributions presented in this study are included in the article. Further inquiries can be directed to the corresponding author.
